# The role of triglyceride-glucose index in the progression of cardiovascular-kidney-metabolic syndrome: findings from the China health and retirement longitudinal study

**DOI:** 10.3389/fcvm.2025.1612121

**Published:** 2025-06-23

**Authors:** Xingyu Zhou, Yisi Liu, Jiafei Peng, Xianliang Zhou, Hongtao Wei

**Affiliations:** ^1^ National Clinical Research Center of Cardiovascular Diseases, State Key Laboratory of Cardiovascular Disease, Fuwai Hospital, National Center for Cardiovascular Diseases, Chinese Academy of Medical Sciences and Peking Union Medical College, Beijing, China; ^2^ Department of Cardiology, Fuwai Hospital, National Center for Cardiovascular Diseases, Chinese Academy of Medical Sciences and Peking Union Medical College, Beijing, China; ^3^ Department of Gastroenterology, Beijing Friendship Hospital, Capital Medical University, Beijing, China; ^4^ State Key Laboratory of Digestive Health, Beijing, China; ^5^ National Clinical Research Center for Digestive Disease, Beijing, China; ^6^ Beijing Key Laboratory of Early Gastrointestinal Cancer Medicine and Medical Devices, Beijing, China

**Keywords:** cardiovascular-kidney-metabolic syndrome, triglyceride-glucose index, CKM progression, metabolic disorders, population heterogeneity

## Abstract

**Background:**

Cardiovascular-kidney-metabolic (CKM) syndrome is highly prevalent among adults and contributes substantially to cardiovascular morbidity and mortality. However, strategies for its early identification and prevention remain inadequately defined. The triglyceride-glucose (TyG) index, a surrogate marker of insulin resistance, may be associated with CKM progression, but its role across diverse populations and CKM progression patterns warrants further investigation.

**Methods:**

A total of 6,311 participants from the China Health and Retirement Longitudinal Study (CHARLS) were included. Associations between the TyG index and CKM progression were assessed using Cox proportional hazards and restricted cubic spline models. Subgroup analyses were conducted by age (<65 vs. ≥65 years), sex, and baseline CKM stage. CKM progression was further categorized to explore associations with specific metabolic outcomes.

**Results:**

Among 6,311 participants (mean age 57.8 years, 46.3% male), 31.3% experienced CKM progression over a median follow-up of 48 months. The TyG index was significantly associated with CKM progression in individuals aged ≥65 years, with a notable interaction between age and TyG (*P* for interaction <0.001). In participants with baseline CKM stage 0 or 1, higher TyG levels predicted greater CKM progression risk. A U-shaped relationship was observed in stage 0 (*P* = 0.018, *P* for non-linearity = 0.09), whereas a linear positive association was noted in stage 1 (*P* = 0.002, *P* for non-linearity = 0.008). Elevated TyG was primarily linked to subsequent hypertriglyceridemia in stage 0 (Q4 vs. Q1: HR 3.13, 95% CI 1.65–5.91, *P* < 0.001) and to future diabetes (Q4 vs. Q1: HR 2.85, 95% CI 1.56–5.22, *P* < 0.01), metabolic syndrome (Q4 vs. Q1: HR 1.62, 95% CI 1.10–2.37, *P* < 0.05), and hypertriglyceridemia (Q3 vs. Q1: HR 1.63, 95% CI 1.13–2.34, *P* < 0.01; Q4 vs. Q1: HR 2.21, 95% CI 1.58–3.08, *P* < 0.01) in stage 1.

**Conclusion:**

Elevated TyG index is a significant predictor of CKM progression, particularly via the development of metabolic abnormalities. Its predictive value varies across age groups and CKM stages. Early screening and intervention targeting TyG levels, especially in older adults and those with early-stage CKM, may be critical for halting CKM progression and reducing future cardiovascular risk.

## Introduction

Cardiovascular disease (CVD), chronic kidney disease (CKD), and metabolic disorders share common pathophysiological mechanisms and frequently co-occur, resulting in a synergistic adverse impact on disease incidence and prognosis (1). With growing recognition of their interrelated pathogenesis and the emergence of novel therapeutic agents targeting multiple pathways simultaneously, the American Heart Association (AHA) introduced the concept of cardiovascular-kidney-metabolic (CKM) syndrome in 2023 (2). This integrative framework emphasizes early detection and intervention to prevent or delay the onset and progression of metabolic dysfunction, CKD, and CVD.

However, data from various countries suggest that a substantial proportion of adults have already progressed to CKM stage 2—characterized by the presence of metabolic syndrome, hypertension, diabetes, hypertriglyceridemia, and/or CKD—by the time of detection (3–5). This reflects the high burden of metabolic dysfunction and underscores the urgency of identifying early-stage risk factors to enable timely intervention. Notably, data from China indicate that more than half of adults are currently classified as CKM stage 2 (6), highlighting the critical need for effective prediction tools and preventive strategies for CKM syndrome.

The triglyceride-glucose (TyG) index, a simple and reliable surrogate marker of insulin resistance (7), has demonstrated predictive value in relation to type 2 diabetes (8), atherosclerosis (9), and cardiovascular events (10, 11). Recent studies have also suggested that TyG-related metabolic indices may be useful in predicting cardiovascular outcomes among patients with CKM syndrome (12, 13). Nevertheless, since clinical CVD represents a late stage in the CKM continuum, the potential role of TyG in predicting earlier stages of CKM progression remains unclear. Moreover, whether this association differs across populations or CKM stages is largely unexplored.

Current evidence on the relationship between TyG and CKM syndrome is limited by several gaps: (a) a lack of large-scale, prospective, population-based studies; (b) insufficient exploration of subgroup differences, particularly by age and sex; and (c) a limited understanding of how TyG influences disease progression across distinct CKM stages, which differ substantially in clinical and metabolic characteristics.

Therefore, using data from the nationally representative China Health and Retirement Longitudinal Study (CHARLS), the present study aimed to evaluate the association between TyG index and CKM syndrome progression. We further examined whether this association varies by age, sex, and baseline CKM stage, in order to better understand the heterogeneity of TyG's prognostic utility in diverse populations.

## Materials and methods

### Study population

The CHARLS is a nationally representative longitudinal cohort study conducted across 450 villages in 150 counties within 28 provinces of China. Initiated by the National School of Development at Peking University, the baseline survey (Wave 1) was conducted between 2011 and 2012, with subsequent follow-up waves in 2013 (Wave 2), 2015 (Wave 3), 2018 (Wave 4), and 2020 (Wave 5). The study protocol was approved by the Institutional Review Board of Peking University (IRB00001052-11015), and all participants provided written informed consent prior to enrollment. The study adhered to the Strengthening the Reporting of Observational Studies in Epidemiology (STROBE) guidelines. Detailed descriptions of the CHARLS methodology have been published previously (14), and the dataset is publicly available upon registration and application via the official website (https://charls.charlsdata.com/).

**Figure 1:**
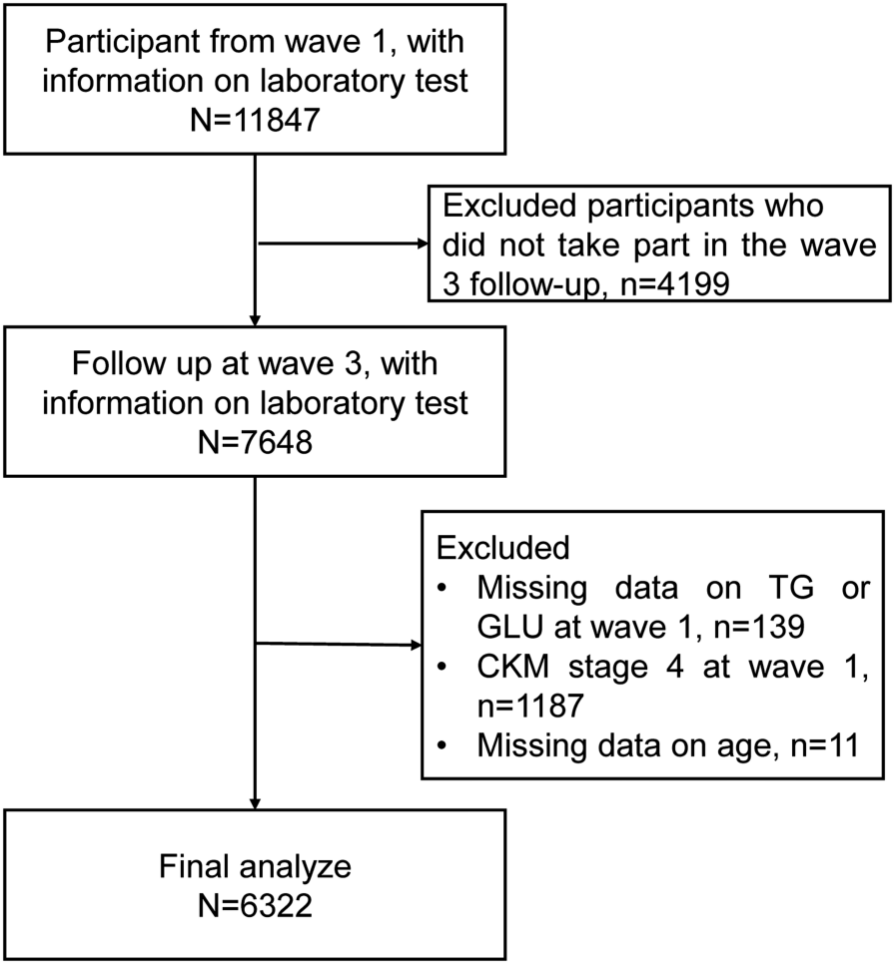
Flowchart illustrating the selection of the study population. TG, triglyceride; GLU, glucose; CKM, cardiovascular-kidney-metabolic syndrome.

The inclusion criteria for this study were as follows: (a) participation in Wave 1 of the CHARLS with available physical examination and blood test data; and (b) availability of follow-up physical and blood test data in Wave 3. Exclusion criteria included: (a) missing data on triglycerides or fasting glucose at baseline (Wave 1); (b) a self-reported history of CVD at or prior to baseline; and (c) missing information on age. A total of 6,311 participants were included in the final analysis. The detailed flowchart of participant selection is presented in Figure 1.

### Data assessment and variable definition

In Wave 1, each participants underwent a standardized in-person interview conducted by trained investigators to collect information on age, sex, education, income, marital status, occupation, smoking, alcohol drinking and medical history. Smoking was categorized as either having a history of smoking or not. Alcohol consumption frequency was divided into less than once a month or more than once a month. CVD history was identified through self-reported medical histories or records.

Anthropometric measurements—including height, weight, waist circumference (WC), and blood pressure (BP)—as well as blood sample collection and assays for blood glucose (GLU), glycosylated hemoglobin (HbA1c), total cholesterol (TC), high-density lipoprotein cholesterol (HDL-C), low-density lipoprotein cholesterol (LDL-C), triglycerides (TG), and serum creatinine (SCR), have been described in detail in the CHARLS cohort profile (14). The estimated glomerular filtration rate (eGFR) was calculated using the CKD Epidemiology Collaboration (CKD-EPI) 2021 creatinine equation (Supplementary Methods) (15).

Hypertension was defined as systolic BP (SBP) ≥140 mmHg, diastolic BP (DBP) ≥90 mmHg, self-reported history of hypertension, or use of anti-hypertensive medications (16, 17). Diabetes was defined as fasting blood glucose levels exceeding 7 mmol/L, random blood glucose levels ≥ 11.1 mmol/L, HbA1c ≥ 6.5%, self-reported history of diabetes, or usage of hypoglycemic agents (18). Dyslipidemia was defined as TC ≥ 6.22 mmol/L, LDL-C ≥ 4.14 mmol/L, HDL-C < 1.04 mmol/L, or TG ≥ 2.26 mmol/L (19). Body mass index (BMI) was calculated as weight in kilograms divided by the square of height in meters. Predicted 10-year CVD risk was calculated based on the PREVENT equation for total 10-year CVD risk (Supplementary Methods) (20, 21), and ≥20% was considered as high predicted 10-year CVD risk. Metabolic syndrome was defined as the presence of 3 or more of the following: (a) WC ≥ 80 cm for women and ≥90 cm for men; (b) HDL-C < 40 mg/dl for men and <50 mg/dl for women; (c) TG ≥ 135 mg/dl; (d) systolic BP (SBP) ≥130 mmHg or diastolic BP (DBP) ≥80 mmHg; and (e) FBG ≥100 mg/dl (1, 2).

Fasting blood glucose (FBG) and TG were collected after a night of fasting and analyzed using a Hitachi 7180 chemistry analyzer (Hitachi, Tokyo, Japan) through Enzymatic colormetric test. The formula of TyG index was: TyG index = ln [TG (mg/dl) × FBG (mg/dl)/2] (11).

### Definition of CKM syndrome and ascertainment of outcome

CKM syndrome was defined and staged from 0 to 4 according to the recommendations of the American Heart Association (AHA) (1, 2). Stage 0 was defined as the absence of any CKM risk factors. Stage 1 included the presence of at least one early risk factor, such as BMI ≥23 kg/m^2^, waist circumference ≥80 cm for women or ≥90 cm for men, FBG between 100 and 124 mg/dl, or HbA1c between 5.7% and 6.4%. Stage 2 was defined by the presence of hypertriglyceridemia (triglycerides ≥135 mg/dl), hypertension, diabetes, metabolic syndrome, and/or CKD. Stage 3 was defined as either advanced CKD (G4 or G5) or a high predicted 10-year CVD risk. Stage 4 was defined as the presence of clinical CVD.

Key variables used for CKM staging were assessed at both Wave 1 and Wave 3. The primary outcome of the study was CKM stage progression, defined as any increase in CKM stage between baseline (Wave 1) and follow-up (Wave 3). Follow-up time was calculated as the difference in integer months between the Wave 3 and Wave 1 survey dates.

### Statistical analysis

Baseline characteristics were summarized according to baseline CKM syndrome stages (Stages 0–3) and outcome status (stability, improvement, or progression). Continuous variables were presented as means with standard deviations (SD) or medians with interquartile ranges (IQR), and differences among groups were assessed using the Kruskal–Wallis test. Categorical variables were presented as counts and percentages, with group comparisons performed using the chi-square test or Fisher's exact test, as appropriate. Missing values for continuous variables were imputed using multiple imputation via the *mice* package in R. The information of missing data is detailed in Supplementary Table S1.

The TyG index was categorized into quartiles (Q1–Q4), with Q1 serving as the reference group. Multivariable Cox proportional hazards regression models were employed to estimate hazard ratios (HRs) and 95% confidence intervals (CIs) for the association between TyG index and CKM progression. Covariate selection for the multivariable models was informed by prior literature and clinical relevance. Model 1 adjusted for age and sex, as both have been shown to significantly influence TyG levels and CKM stage distributions. Model 2 further included key socioeconomic factors—annual household income, occupation status, educational level, marital status, and urban/rural residence—based on recent studies demonstrating their impact on CKM stage (22). In Model 3, additional adjustments were made for smoking status, alcohol consumption, baseline CKM stage, and LDL-C levels. Smoking and alcohol use are established risk factors for cardiovascular disease progression. Baseline CKM stage was included to account for underlying metabolic and renal status; however, since LDL-C is not part of the CKM staging criteria yet plays an important role in cardiovascular risk, it was included separately in the fully adjusted model. We conducted collinearity diagnostics for all covariates in Model 3, and all variance inflation factors (VIF) were below 5 (Supplementary Table S2), indicating no multicollinearity among the covariates. The proportional hazards assumption was evaluated using Schoenfeld residuals, with no significant violations observed. Restricted cubic spline (RCS) regression, based on the Cox model, was used to explore potential nonlinear associations between the TyG index and CKM progression.

Subgroup analyses were conducted by age (<65 vs. ≥65 years), sex, and baseline CKM stage. All subgroup models were adjusted for age, sex, annual household income, occupation status, educational level, marital status, urbanity, smoking, alcohol consumption, and LDL-C.

A two-sided *P* value <0.05 was considered statistically significant. All analyses were performed using Stata version 18 and R version 4.3.1.

## Results

### Participant characteristics

A total of 6,311 participants with an average age of 57.8 ± 9.1 years were included in this study, with 46.3% being male, 91.2% having education level of less than lower secondary education, 89.9% currently married, 25.2% earning more than 10,000 Chinese Yuan annually, 70.5% being employed, and 66.9% residing in rural areas (Table 1). Regarding metabolic risk factors and comorbidities, 44.8% had hypertension, 13.8% had diabetes, 9.0% had dyslipidemia, 13.9% had obesity (BMI ≥ 28.0 kg/m^2^), 8.6% had predicted 10-year CVD risk ≥20%, 29.7% currently smoking, 25.0% currently consuming alcohol more than once a month (Table 1).

**Table 1:** Baseline characteristic.

Characteristic	Total	CKM stages				*P* value
		Stage 0	Stage 1	Stage 2	Stage 3	
*N* (%)	6,311 (100)	671 (10.6)	1,323 (21.0)	3,837 (60.8)	480 (7.6)	NA
Age, years, mean (SD)	57.8 (9.1)	56.8 (8.5)	55.5 (8.0)	57.0 (8.1)	71.8 (7.9)	<0.001
Sex, (%)						
Male	2,921 (46.3)	402 (59.9)	578 (43.7)	1,624 (42.3)	317 (66.0)	<0.001
Female	3,390 (53.7)	269 (40.1)	745 (56.3)	2,213 (57.7)	163 (34.0)	
Education level, (%)						
Less than lower secondary education	5,757 (91.2)	606 (90.3)	1,205 (91.1)	3,488 (90.9)	458 (95.4)	0.005
Upper secondary & vocational training	498 (7.9)	62 (9.2)	104 (7.9)	316 (8.2)	16 (3.3)	
Tertiary education	56 (0.9)	3 (0.5)	14 (1.1)	33 (0.9)	6 (1.3)	
Married, (%)	5,673 (89.9)	620 (92.4)	1,216 (91.9)	3,483 (90.8)	354 (73.8)	<0.001
Household income >10,000 yuan/year, (%)	1,592 (25.2)	116 (17.3)	341 (25.8)	1,047 (27.3)	88 (18.3)	<0.001
Employed, (%)	4,448 (70.5)	524 (78.1)	1,018 (77.0)	2,689 (70.1)	217 (45.2)	<0.001
Urbanity, (%)						
Rural	4,233 (66.9)	499 (74.4)	924 (69.8)	2,482 (64.7)	318 (66.3)	<0.001
Urban	2,088 (33.1)	172 (25.6)	399 (30.2)	1,355 (35.3)	162 (33.8)	
Metabolic risk factors and complications						
Hypertension, (%)	2,830 (44.8)	NA	NA	2,459 (64.1)	371 (77.3)	<0.001
Diabetes, (%)	873 (13.8)	NA	NA	674 (17.6)	199 (41.5)	<0.001
Dyslipidemia, (%)	565 (9.0)	14 (2.1)	67 (5.1)	426 (11.1)	58 (12.1)	<0.001
Obesity, (%)	612 (9.7)	NA	80 (6.1)	480 (12.5)	52 (10.8)	<0.001
BMI, kg/m^2^, mean (SD)	23.9 (13.9)	20.2 (1.7)	23.1 (3.5)	25.1 (18.3)	23.3 (4.6)	<0.001
CVD risk, mean (SD)	8.6 (5.1)	5.8 (4.5)	4.6 (3.7)	8.2 (5.1)	25.8 (5.1)	<0.001
High 10-year CVD risk, (%)	480 (8.6)	NA	NA	NA	480 (100)	NA
Waist, cm, mean (SD)	84.1 (12.3)	74.7 (9.0)	82.1 (11.1)	86.6 (12.0)	86.1 (13.4)	<0.001
SBP, mmHg, mean (SD)	128.2 (20.9)	114.3 (11.6)	115.4 (10.8)	133.5 (20.2)	148.6 (25.1)	<0.001
DBP, mmHg, mean (SD)	74.9 (12.1)	67.9 (8.9)	69.0 (8.2)	78.5 (12.1)	78.3 (14.0)	<0.001
Current alcohol drinking, (%)	1,579 (25.0)	208 (31.0)	320 (24.2)	912 (23.8)	139 (29.0)	<0.001
Current smoker, (%)	1,873 (29.7)	280 (41.7)	360 (27.2)	982 (25.6)	251 (52.3)	<0.001
Triglyceride-glucose index related data						
TG, mg/dl	133.1 (110.8)	78.2 (25.7)	83.9 (24.8)	156.3 (116.7)	159.9 (184.5)	<0.001
GLU, mg/dl	109.4 (34.9)	91.6 (11.4)	100.7 (11.3)	113.1 (36.9)	128.4 (58.9)	<0.001
Triglyceride-glucose index	8.7 (0.7)	8.1 (0.4)	8.3 (0.3)	8.9 (0.7)	8.9 (0.8)	<0.001
Laboratory test						
TC, mg/dl	192.5 (38.1)	180.1 (31.8)	188.5 (35.1)	196.2 (38.9)	191.1 (42.0)	<0.001
LDL-C, mg/dl	115.4 (34.4)	108.6 (28.0)	116.5 (30.7)	116.7 (36.1)	111.7 (36.1)	<0.001
HDL-C, mg/dl	51.2 (15.3)	58.3 (15.0)	57.3 (14.0)	48.3 (14.9)	47.4 (14.9)	<0.001
SCR, mg/dl	0.8 (0.2)	0.8 (0.2)	0.7 (0.2)	0.8 (0.2)	0.9 (0.3)	<0.001
eGFR, ml/min/1.73 m^2^	97.6 (13.4)	100.3 (11.3)	100.5 (11.4)	97.8 (13.1)	84.4 (15.6)	<0.001
HbA1c, %	5.2 (0.8)	5.0 (0.3)	5.1 (0.4)	5.3 (0.9)	5.5 (1.2)	<0.001

*N*, number; SD, standard deviation; BMI, body mass index; CVD, cardiovascular disease; SBP, systolic blood pressure; DBP, diastolic blood pressure; TG, triacylglycerol; GLU, blood glucose; TC, total cholesterol; LDL-C, low-density lipoprotein cholesterol; HDL-C, high-density lipoprotein cholesterol; SCR, serum creatinine; eGFR, estimated glomerular filtration rate; HbA1c, glycated hemoglobin. NA, not available. Categorical variables were presented as number (percentage) and compared using the Pearson *χ*^2^ test. Continuous variables were displayed as mean (SD), and compared using Kruskal–Wallis tests.

The prevalence of CKM syndrome at baseline was 89.4%, with 21.0% at stage 1, 60.8% at stage 2, and 7.6% at stage 3. Male participants, those who were older, with lower education level, urban residents were more likely to exhibit higher CKM stages, while those who were married, with higher income, and employed were more likely to exhibit lower CKM stages (both *P* for trend <0.001) (Table 1). The presence of hypertension, diabetes, dyslipidemia, obesity, and higher TyG index were associated with higher CKM stages (both *P* for trend <0.001) (Table 1).

### Distribution of baseline variables across CKM progression, improvement, and stability groups

After a median follow-up of 48 months (IQR, 47–48), 56.4% of participants remained at the same CKM stage, 12.5% showed improvement, and 31.1% experienced progression. CKM progression was more common among males and older participants, while those who were employed had a higher prevalence of CKM stage improvement. In contrast, individuals with hypertension, diabetes, or dyslipidemia were more likely to maintain a stable CKM stage (Table 2).

**Table 2:** Comparison of baseline characteristics across CKM syndrome progression categories.

Characteristic	CKM stages variation			*P* value
	Stability	Improvement	Progression	
*N* (%)	3,558 (56.4)	790 (12.5)	1,963 (31.1)	NA
Age, years, mean (SD)	57.3 (9.1)	56.3 (8.2)	59.0 (9.2)	<0.001
Sex, (%)				
Male	1,560 (43.8)	396 (50.13)	965 (49.2)	<0.001
Female	1,998 (56.2)	394 (49.9)	998 (50.8)	
Education level, (%)				
Less than lower secondary education	3,235 (90.9)	724 (91.7)	1,798 (91.6)	0.25
Upper secondary & vocational training	285 (8.0)	64 (8.1)	149 (7.6)	
Tertiary education	38 (1.1)	2 (0.3)	16 (0.8)	
Married, (%)	3,221 (90.5)	721 (91.3)	1,731 (88.2)	0.008
Household income >10,000 yuan/year, (%)	934 (26.3)	201 (25.4)	457 (23.3)	0.051
Employed, (%)	2,537 (71.3)	569 (72.0)	1,342 (68.4)	0.04
Urbanity, (%)				
Rural	2,347 (66.0)	532 (67.3)	1,344 (68.5)	0.16
Urban	1,211 (34.0)	258 (32.7)	619 (31.5)	
Metabolic risk factors and complications				
Hypertension, (%)	1,811 (50.9)	385 (48.7)	634 (32.3)	<0.001
Diabetes, (%)	591 (16.6)	107 (13.5)	175 (8.9)	<0.001
Dyslipidemia, (%)	351 (9.9)	45 (5.7)	169 (8.6)	0.001
CVD risk, mean (SD)	8.9 (7.6)	8.4 (7.6)	8.2 (6.4)	0.03
BMI, kg/m^2^, mean (SD)	24.3 (12.8)	22.7 (4.1)	23.5 (17.5)	<0.001
Waist, cm, mean (SD)	85.3 (12.2)	81.7 (11.3)	82.6 (12.4)	<0.001
SBP, mmHg, mean (SD)	130.2 (21.5)	128.0 (21.8)	124.7 (19.0)	<0.001
DBP, mmHg, mean (SD)	76.2 (12.5)	74.9 (12.1)	72.7 (10.9)	<0.001
Triglyceride-glucose index	8.8 (0.7)	8.7 (0.7)	8.5 (0.6)	<0.001
Current alcohol drinking, (%)	848 (23.8)	209 (26.5)	522 (26.6)	0.047
Current smoker, (%)	988 (27.8)	263 (33.3)	622 (31.7)	0.001
Laboratory test				
GLU, mg/dl	112.3 (38.6)	108.4 (29.0)	104.4 (28.8)	<0.001
TC, mg/dl	194.7 (38.5)	186.6 (38.7)	191.0 (36.7)	<0.001
LDL-C, mg/dl	116.3 (35.3)	109.1 (32.7)	116.3 (33.0)	<0.001
HDL-C, mg/dl	49.8 (15.1)	51.0 (15.1)	53.9 (15.5)	<0.001
TG, mg/dl	144.5 (115.4)	137.2 (147.2)	110.9 (76.7)	<0.001
SCR, mg/dl	0.8 (0.2)	0.8 (0.2)	0.8 (0.2)	0.07
eGFR, ml/min/1.73 m^2^	97.6 (13.5)	100.1 (12.0)	96.5 (13.5)	<0.001
HbA1c, %	5.3 (0.9)	5.1 (0.6)	5.2 (0.7)	<0.001

SD, standard deviation; CVD, cardiovascular disease; BMI, body mass index; SBP, systolic blood pressure; DBP, diastolic blood pressure; GLU, blood glucose; TC, total cholesterol; LDL-C, low-density lipoprotein cholesterol; HDL-C, high-density lipoprotein cholesterol; TG, triacylglycerol; SCR, serum creatinine; eGFR, estimated glomerular filtration rate; HbA1c, glycated hemoglobin; NA, not available. Categorical variables were presented as number (percentage) and compared using the Pearson *χ*^2^ test. Continuous variables were displayed as mean (SD), and compared using Kruskal–Wallis tests.

Participants with lower baseline CKM stages were more likely to experience CKM progression, with progression rates of 76.3%, 48.4%, 12.7%, and 11.3% for baseline CKM stages 0–3, respectively. Most cases of progression occurred to the adjacent CKM stage (Table 3). Conversely, those with higher baseline CKM stages were more likely to remain at the same stage during follow-up (Table 3).

**Table 3:** The progress of CKM stages.

CKM stage	Baseline			
Follow-up visit	Stage 0, *n* = 671	Stage 1, *n* = 1,323	Stage 2, *n* = 3,827	Stage 3, *n* = 480
Stage 0, *n* = 379	159 (23.7)	102 (7.7)	114 (3.0)	4 (0.8)
Stage 1, *n* = 1,342	254 (37.9)	581 (43.9)	499 (13.0)	8 (1.7)
Stage 2, *n* = 3,221	175 (26.1)	516 (39.0)	2,467 (64.3)	63 (13.1)
Stage 3, *n* = 793	49 (7.3)	46 (3.5)	347 (9.0)	351 (73.1)
Stage 4, *n* = 576	34 (5.1)	78 (5.9)	410 (10.7)	54 (11.3)

CKM, cardiovascular-kidney-metabolic syndrome. Data were presented as number (percentage).

### Association between TyG index and CKM progression

**Figure 2:**
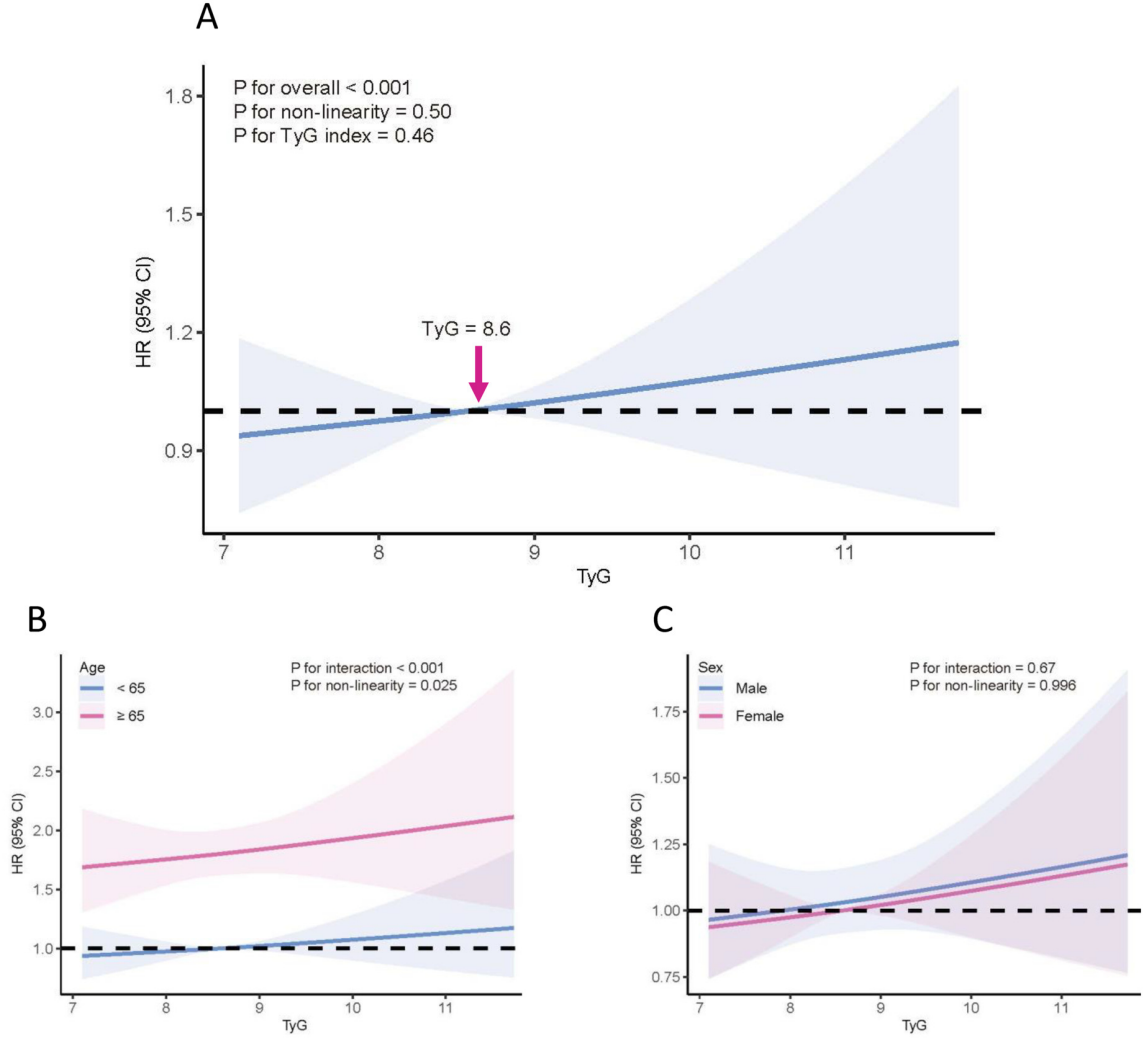
Association between TyG index and CKM progression. **(A)** Overall population; **(B)** stratified by age group; **(C)** stratified by sex. The solid red line represents the estimated hazard ratio (HR), and the blue-shaded area indicates the 95% confidence interval (CI). Models were adjusted for age, sex, annual household income, marital status, employment status, education level, urbanity, smoking, drinking, LDL-C, and baseline CKM stage. CKM, cardiovascular-kidney-metabolic; TyG, triglyceride-glucose index; HR, hazard ratio; CI, confidence interval.

In Model 1, only age and sex were adjusted; Model 2 further adjusted for socioeconomic factors. Both models showed negative association between TyG and CKM progression (Supplementary Figure S1). In model 3, we further adjusted for smoking, drinking, baseline CKM stage, and LDL-C, the nonlinearity test for the TyG index was not statistically significant (*P* for non-linearity = 0.50), and its overall association with CKM progression was also not significant (*P* = 0.46), although a positive trend was observed (Figure 2A). In subgroup analyses, the association between TyG and CKM progression varied by age group. Among participants aged >65 years, TyG was significantly associated with an increased risk of CKM progression, with evidence of a nonlinear relationship (*P* for non-linearity = 0.025). A significant interaction was observed between TyG and age (*P* for interaction <0.001) (Figure 2B). However, no significant interaction was found between TyG and sex (*P* for interaction = 0.67) (Figure 2C). Moreover, a significant interaction was observed between TyG levels and baseline CKM stage (*P* for interaction <0.001). Therefore, subgroup analyses were subsequently conducted according to baseline CKM stage.

When TyG was analyzed as a categorical variable (IQR), no significant association was observed with CKM progression in most comparisons (Supplementary Table S3).

### Association between TyG index and CKM progression across different CKM stages

**Figure 3:**
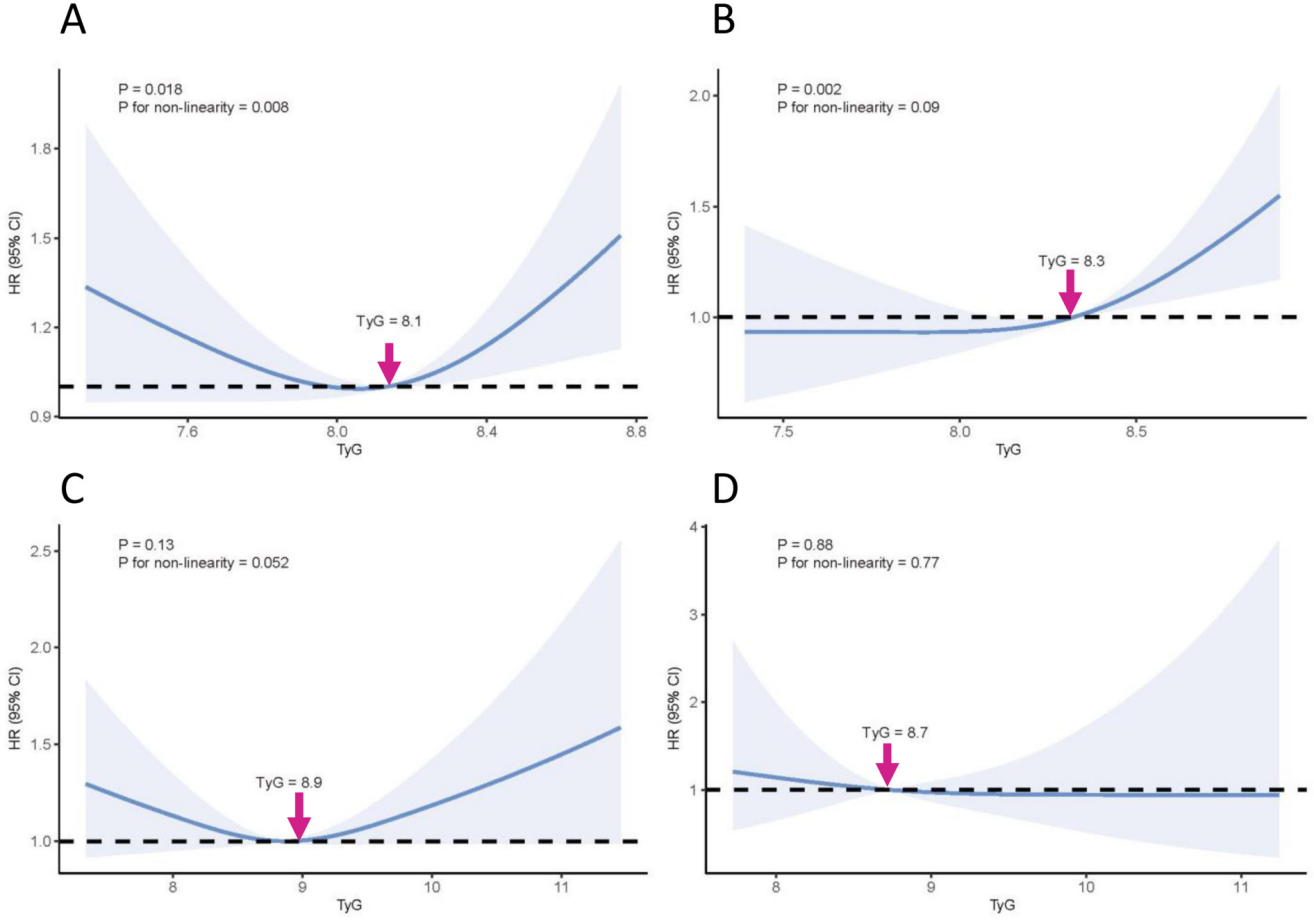
Association between TyG index and CKM progression across different CKM stages. **(A)** CKM stage 0; **(B)** CKM stage 1; **(C)** CKM stage 2; **(D)** CKM stage 3. The solid red line represents the estimated hazard ratio (HR), and the blue-shaded area indicates the 95% confidence interval (CI). Models were adjusted for age, sex, annual household income, marital status, employment status, education level, urbanity, smoking, drinking, LDL-C, and baseline CKM stage. CKM, cardiovascular-kidney-metabolic; TyG, triglyceride-glucose index; HR, hazard ratio; CI, confidence interval.

The association between the TyG index and CKM progression varied across different baseline CKM stages. Among participants with baseline CKM stage 0, a U-shaped relationship was observed between the TyG index and CKM progression (*P* for non-linearity = 0.018). When the TyG index was ≥8.1, the risk of CKM progression increased significantly with increasing TyG levels (Figure 3A). For those at CKM stage 1, a positive association was observed between TyG and CKM progression (*P* for non-linearity = 0.09); the risk of progression increased markedly when TyG was ≥8.3 (Figure 3B). In participants with CKM stage 2, a U-shaped pattern was noted; however, the overall association was not statistically significant (*P* > 0.05) (Figure 3C). For those at CKM stage 3, no significant association was observed between the TyG index and CKM progression (Figure 3D).

Given that the TyG index was significantly associated with CKM progression only in patients with baseline CKM stage 0 and 1, we further examined specific progression patterns in these subgroups. Outcomes included: (1) progression to CKM stage 2 components (i.e., hypertension, diabetes, hypertriglyceridemia, metabolic syndrome, or CKD); (2) incident CVD; (3) intertemporal CKM progression (defined as CKM stage increase ≥2 between wave 1 and wave 3); and (4) CKM stage increase by 1. As shown in Table 4, the multivariate adjustment model shows in individuals with CKM stage 0, elevated TyG index significantly increased the risk of future hypertriglyceridemia (Q4 vs. Q1: HR 3.13, 95% CI 1.65–5.91), contributing to CKM progression. In those with CKM stage 1, higher TyG levels were significantly associated with subsequent development of diabetes (Q4 vs. Q1: HR 2.85, 95% CI 1.56–5.22), metabolic syndrome (Q4 vs. Q1: HR 1.62, 95% CI 1.10–2.37), and hypertriglyceridemia (Q3 vs. Q1: HR 1.63, 95% CI 1.13–2.34; Q4 vs. Q1: HR 2.21, 95% CI 1.58–3.08), leading to further progression of CKM. However, no significant association was found between TyG levels and CKM stage progression defined as an increase of two or more stages, as well as with CKD, or clinical CVD (Supplementary Table S4).

**Table 4:** The HR and 95%CI for the association of TyG index levels and the progress of CKM stages among participants with CKM stages 0 and 1 at wave 1.

Different types of CKM progression	CKM stage 0 at wave 1			CKM stage 1 at wave 1		
	Model 1	Model 2	Model 3	Model 1	Model 2	Model 3
Hypertension						
Q1	Ref.	Ref.	Ref.	Ref.	Ref.	Ref.
Q2	0.88 (0.48, 1.61)	0.91 (0.49, 1.70)	0.99 (0.53, 1.87)	0.65 (0.43, 0.99)*	0.64 (0.42, 0.97)*	0.59 (0.39, 0.91)*
Q3	1.01 (0.60, 1.72)	1.03 (0.61, 1.77)	0.94 (0.55, 1.63)	1.02 (0.71, 1.46)	0.97 (0.67, 1.40)	0.93 (0.64, 1.36)
Q4	0.89 (0.51, 1.54)	0.99 (0.56, 1.73)	0.86 (0.47, 1.59)	0.87 (0.62, 1.23)	0.84 (0.60, 1.19)	0.74 (0.51, 1.07)
Diabetes						
Q1	Ref.	Ref.	Ref.	Ref.	Ref.	Ref.
Q2	1.60 (0.61, 4.15)	1.76 (0.66, 4.66)	1.80 (0.65, 4.98)	1.73 (0.88, 3.38)	1.65 (0.84, 3.24)	1.64 (0.83, 3.24)
Q3	1.91 (0.47, 3.03)	1.24 (0.49, 3.17)	1.16 (0.44, 3.05)	1.70 (0.88, 3.30)	1.56 (0.80, 3.05)	1.70 (0.86, 3.36)
Q4	0.85 (0.31, 2.35)	0.84 (0.30, 2.35)	0.80 (0.26, 2.43)	2.89 (1.62, 5.15)***	2.76 (1.55, 4.94)**	2.85 (1.56, 5.22)**
Hypertriglyceridemia						
Q1	Ref.	Ref.	Ref.	Ref.	Ref.	Ref.
Q2	1.37 (0.65, 2.88)	1.32 (0.62, 2.78)	1.31 (0.61, 2.80)	1.18 (0.79, 1.76)	1.24 (0.83, 1.85)	1.16 (0.77, 1.73)
Q3	1.61 (0.83, 3.14)	1.70 (0.87, 3.32)	1.56 (0.78, 3.09)	1.75 (1.22, 2.50)**	1.81 (1.27, 2.59)**	1.63 (1.13, 2.34)**
Q4	3.72 (2.06, 6.72)***	3.71 (2.04, 6.75)***	3.13 (1.65, 5.91)***	2.49 (1.81, 3.42)***	2.62 (1.90, 3.61)***	2.21 (1.58, 3.08)***
Metabolic syndrome						
Q1	Ref.	Ref.	Ref.	Ref.	Ref.	Ref.
Q2	0.57 (0.18, 1.81)	0.47 (0.15, 1.52)	0.49 (0.15, 1.61)	0.99 (0.63, 1.58)	0.99 (0.62, 1.57)	0.91 (0.57, 1.46)
Q3	0.58 (0.21, 1.59)	0.63 (0.23, 1.74)	0.44 (0.15, 1.28)	1.61 (1.08, 2.42)*	1.63 (1.09, 2.45)*	1.38 (0.91, 2.09)
Q4	2.67 (1.29, 5.51)**	2.41 (1.16, 5.02)*	1.52 (0.69, 3.38)	2.06 (1.43, 2.96)***	2.11 (1.46, 3.05)***	1.62 (1.10, 2.37)*
CKD						
Q1	Ref.	Ref.	Ref.	Ref.	Ref.	Ref.
Q2	0.66 (0.06, 7.24)	1.11 (0.08, 14.56)	1.25 (0.07, 21.60)	2.48 (0.93, 6.62)	2.19 (0.81, 5.91)	2.39 (0.85, 6.70)
Q3	1.47 (0.25, 8.82)	1.84 (0.28, 12.03)	3.24 (0.38, 27.77)	1.71 (0.61, 4.82)	1.70 (0.60, 4.82)	2.00 (0.68, 5.88)
Q4	2.34 (0.45, 12.11)	2.30 (0.38, 13.91)	2.87 (0.27, 30.28)	1.65 (0.62, 4.40)	1.55 (0.58, 4.16)	1.80 (0.63, 5.13)
Clinical CVD						
Q1	Ref.	Ref.	Ref.	Ref.	Ref.	Ref.
Q2	0.63 (0.22, 1.82)	0.69 (0.23, 2.00)	0.67 (0.22, 1.99)	1.01 (0.51, 1.98)	0.87 (0.44, 1.73)	0.84 (0.42, 1.69)
Q3	0.45 (0.16, 1.29)	0.43 (0.15, 1.26)	0.36 (0.12, 1.08)	1.59 (0.88, 2.90)	1.41 (0.77, 2.58)	1.40 (0.75, 2.62)
Q4	1.18 (0.53, 2.65)	1.35 (0.59, 3.07)	1.10 (0.45, 2.68)	0.87 (0.46, 1.64)	0.76 (0.40, 1.46)	0.71 (0.36, 1.40)
CKM stage at wave 3—CKM stage at wave 1 ≥ 2						
Q1	Ref.	Ref.	Ref.	Ref.	Ref.	Ref.
Q2	1.01 (0.68, 1.48)	1.04 (0.70, 1.54)	1.06 (0.71, 1.58)	1.09 (0.64, 1.87)	0.93 (0.54, 1.61)	0.85 (0.49, 1.48)
Q3	1.06 (0.75, 1.51)	1.11 (0.78, 1.58)	1.04 (0.73, 1.49)	1.39 (0.85, 2.29)	1.14 (0.69, 1.88)	1.12 (0.67, 1.88)
Q4	1.40 (1.01, 1.94)*	1.46 (1.04, 2.04)*	1.32 (0.93, 1.89)	1.06 (0.65, 1.73)	0.88 (0.54, 1.46)	0.77 (0.45, 1.30)
CKM stage at wave 3—CKM stage at wave 1 = 1						
Q1	Ref.	Ref.	Ref.	Ref.	Ref.	Ref.
Q2	0.81 (0.56, 1.16)	0.80 (0.56, 1.16)	0.75 (0.52, 1.09)	1.04 (0.79, 1.37)	1.06 (0.80, 1.40)	1.02 (0.77, 1.35)
Q3	0.87 (0.63, 1.21)	0.85 (0.61, 1.18)	0.82 (0.59, 1.14)	1.31 (1.01, 1.69)*	1.35 (1.04, 1.74)*	1.28 (0.98, 1.67)
Q4	0.80 (0.57, 1.11)	0.79 (0.56, 1.11)	0.77 (0.54, 1.10)	1.49 (1.18, 1.88)***	1.54 (1.22, 1.95)***	1.40 (1.09, 1.79)**

TyG, triglyceride-glucose index; CKM, cardiovascular-kidney-metabolic syndrome. Data were presented as number (percentage); SD, standard deviation; HR, hazard ratio, CI confidence intervals. Cox proportional hazards regression models were employed to estimate hazard ratio and 95% confidence intervals for the association between TyG index levels and CKM progression. Model 1: non-adjusted; Model 2: adjusted for age, gender, marriage status, annuals household income, occupation status, education levels, urbanity, smoking, alcohol drinking; Model 3: adjusted for for age, gender, marriage status, annuals household income, occupation status, education levels, urbanity, smoking, alcohol drinking, low-density lipoprotein cholesterol, eGFR, BMI, waist, HbA1c, HDL-C, SBP, and DBP.

*** *P* < 0.001.

** *P* < 0.01.

* *P* < 0.05.

## Discussion

In this study, the association between the TyG index and the progression of CKM syndrome was significantly modified by age and baseline CKM stage. Although no significant association was observed in the overall population, a positive relationship emerged among individuals aged ≥65 years. RCS analysis further demonstrated that the TyG index was significantly associated with CKM progression in participants at baseline CKM stage 0 and 1. Specifically, a U-shaped association was observed at CKM stage 0, whereas a linear positive association was evident at stage 1. In contrast, no significant associations were found between TyG index and CKM progression among individuals with baseline CKM stage 2 or 3.

Consistent with previous studies (3, 5, 12), our findings revealed that the majority of adults were already at CKM stage 2 at the time of baseline screening. Moreover, we observed that the proportion of individuals experiencing CKM stage progression exceeded that of those demonstrating improvement, with lower baseline CKM stages being associated with a higher likelihood of progression. During a median follow-up of approximately 2 years, most individuals at baseline CKM stage 0 progressed to stage 1, followed by stage 2; those initially at stage 1 primarily progressed to stage 2. Notably, a substantial proportion of individuals at stage 2 remained at that stage, suggesting a shift from normal to abnormal metabolic states or worsening metabolic profiles during follow-up. However, relatively few individuals progressed to advanced CKM (stages 3–4). Metabolic abnormalities were more prevalent than CVD events, indicating that metabolic dysregulation was the dominant component of CKM progression. The triglyceride-glucose (TyG) index may serve as a valuable predictive biomarker for metabolic dysfunction, as it has been previously associated with insulin resistance (8, 23), diabetes (24), hypertension (25), metabolic syndrome (26), CKD (27, 28), and CVD (10, 11).

Previous studies have shown that although the triglyceride-glucose (TyG) index is associated with incident CVD across CKM stages 0–3, its short-term predictive performance is suboptimal, with a C-index <0.6 for events occurring within 3 years (12). Additionally, the TyG index has been reported to be significantly associated with CVD mortality but not with all-cause mortality in patients with CKM (13). Data specifically exploring the association between TyG index and CKM syndrome remain limited. In our study, Models 1 and 2 demonstrated a negative association between the TyG index and CKM progression. However, after adjusting for baseline CKM stage and additional covariates in Model 3, this association reversed direction and became positive, while the test for non-linearity was no longer significant. This change likely reflects the strong influence of baseline CKM stage, which is a well-established predictor of disease progression. Including baseline CKM stage in the model may have attenuated or modified the apparent effect of TyG, as it captures a substantial portion of the risk attributed to metabolic abnormalities. Consequently, the independent association between TyG and CKM progression appears to be weaker and more complex than initially observed. These findings underscore the importance of accounting for baseline disease severity when evaluating the prognostic value of metabolic indices such as TyG. It also suggests that TyG may have limited incremental predictive value beyond established clinical indicators. Future research should focus on longitudinal analyses examining dynamic changes in TyG and CKM stage to better clarify their joint effects on progression risk. When TyG was analyzed as a categorical variable (IQR), its association with CKM progression was largely non-significant, which may reflect information loss and reduced statistical power resulting from categorizing a continuous variable, highlighting the advantage of modeling TyG as a continuous measure to better capture potential dose–response relationships. In subgroup analysis, a significant positive association between the TyG index and CKM progression was observed among the elderly population (aged ≥65 years), accompanied by a significant interaction between TyG index and age. This finding may be attributed to the close link between elevated TyG levels and increased biological aging, which is associated with heightened susceptibility to metabolic dysregulation and accelerated cardiovascular and renal systems deterioration in older adults (29).

Previous studies have reported that TyG-related indices (e.g., TyG-BMI, TyG-WC, TyG-WHtR), rather than the TyG index alone, are associated with CVD incidence and mortality in individuals with CKM syndrome (12, 13, 30), suggesting that TyG may influence CVD risk primarily through its association with metabolic disorders. Building on this, our study first demonstrated significant stage-specific heterogeneity in the association between TyG and CKM progression, with significant effects observed only in CKM stage 0 and 1. A U-shaped association between TyG index and CKM progression was observed in individuals at CKM stage 0, likely due to the inclusion of metabolically healthy individuals with low BMI and glucose levels. Previous studies have reported associations between low BMI (31, 32) and hypoglycemia (33, 34) increased risk of CVD related events. In contrast, among CKM stage 1 individuals, TyG index was positively associated with CKM progression, highlighting its predictive value for disease progression in populations with obesity or prediabetes. Furthermore, among various types of CKM progression, elevated TyG was primarily associated with an increased risk of future diabetes, metabolic syndrome, and hypertriglyceridemia, while its direct association with CVD progression was not statistically significant. These findings highlight the potential utility of the TyG index in predicting early metabolic deterioration in CKM and support the need for timely intervention in individuals with elevated TyG to mitigate future cardiometabolic risk.

The TyG index, as a surrogate marker of insulin resistance, may promote CKM progression through multiple biological pathways. Insulin resistance contributes to endothelial dysfunction, chronic inflammation, and oxidative stress, all of which are implicated in the pathogenesis of both cardiovascular and renal dysfunctions (35, 36). Elevated TyG levels have also been associated with early arterial stiffness and subclinical atherosclerosis, which may accelerate the progression of metabolic and cardiorenal diseases (37). Our study underscoring the urgent need for enhanced CKM prevention and control strategies. Early identification and timely intervention targeting metabolic risk factors are critical to halting CKM progression.

This study yields several important implications. First, it fills a critical gap in the literature by elucidating the association between the TyG index and the overall progression of CKM syndrome. Second, it highlights the heterogeneity in TyG-related risk across different CKM stages, underscoring the necessity for stage-specific risk stratification and management strategies. Third, the TyG index was a strong predictor of key metabolic abnormalities—including diabetes, metabolic syndrome, and hypertriglyceridemia—supporting the hypothesis that TyG may increase CVD morbidity and mortality primarily through its impact on metabolic dysregulation. These findings suggest that in early-stage CKM (stages 0 and 1), proactive screening, monitoring, and intervention targeting TyG may be valuable for preventing or delaying CKM progression and reducing long-term cardiovascular risk.

This study has several limitations. First, due to its observational nature, the potential for residual confounding remains, limiting the ability to infer causality from observed associations. Second, CVD were obtained from self-reported medical histories, which may be subject to recall bias, although this approach is consistent with previous studies. Third, the absence of detailed clinical indicators—such as coronary angiography or CT angiography findings, N-terminal pro-B-type natriuretic peptide, high-sensitivity troponin T, and echocardiographic parameters—may restrict the comprehensive assessment of sub-clinical CVD. Fourth, the CHARLS cohort primarily includes individuals aged 45 years and older, limiting the generalizability of the findings to younger populations and other ethnic groups. Future research should aim to validate these results in more diverse and clinically detailed cohorts. Fifth, subgroup analyses based on specific metabolic components within the CKM framework (e.g., diabetes, hypertension, dyslipidemia) were not conducted in this study. Future research should explore whether individual components exert differential impacts on CKM progression and outcomes. Finally, the 48 months follow-up may limit assessment of long-term CKD and CVD risk, warranting validation in longer studies.

## Conclusion

The TyG index demonstrates prognostic utility for CKM progression among individuals of advanced age and those at CKM stages 0 and 1, particularly in predicting the onset of metabolic disorders. Early screening, continuous monitoring, and timely intervention targeting elevated TyG levels in these populations may aid in preventing or delaying the development of metabolic abnormalities, thereby contributing to the long-term reduction of CVD incidence and mortality.

## Data Availability

The original contributions presented in the study are included in the article/Supplementary Material, further inquiries can be directed to the corresponding authors.

## References

[1] Ndumele CE Neeland IJ Tuttle KR Chow SL Mathew RO Khan SS A synopsis of the evidence for the science and clinical management of cardiovascular-kidney-metabolic (CKM) syndrome: a scientific statement from the American heart association. Circulation. (2023) 148(20):1636–64. 10.1161/cir.0000000000001186 37807920

[2] Ndumele CE Rangaswami J Chow SL Neeland IJ Tuttle KR Khan SS Cardiovascular-kidney-metabolic health: a presidential advisory from the American heart association. Circulation. (2023) 148(20):1606–35. 10.1161/cir.0000000000001184 37807924

[3] Aggarwal R Ostrominski JW Vaduganathan M. Prevalence of cardiovascular-kidney-metabolic syndrome stages in US adults, 2011-2020. J Am Med Assoc. (2024) 331(21):1858–60. 10.1001/jama.2024.6892 PMC11079779 38717747

[4] Minhas AMK Mathew RO Sperling LS Nambi V Virani SS Navaneethan SD Prevalence of the cardiovascular-kidney-metabolic syndrome in the United States. J Am Coll Cardiol. (2024) 83(18):1824–6. 10.1016/j.jacc.2024.03.368 38583160

[5] Yim Y Lee JE Son Y Kim S Lee H Lee S Long-term trends in the prevalence of cardiovascular-kidney-metabolic syndrome in South Korea, 2011-2021: a representative longitudinal serial study. Lancet Reg Health West Pac. (2025) 55:101474. 10.1016/j.lanwpc.2025.101474 39911647 PMC11795540

[6] Li N Li Y Cui L Shu R Song H Wang J Association between different stages of cardiovascular-kidney-metabolic syndrome and the risk of all-cause mortality. Atherosclerosis. (2024) 397:118585. 10.1016/j.atherosclerosis.2024.118585 39255681

[7] Minh HV Tien HA Sinh CT Thang DC Chen CH Tay JC Assessment of preferred methods to measure insulin resistance in Asian patients with hypertension. J Clin Hypertens. (2021) 23(3):529–37. 10.1111/jch.14155 PMC8029536 33415834

[8] Guerrero-Romero F Simental-Mendía LE González-Ortiz M Martínez-Abundis E Ramos-Zavala MG Hernández-González SO The product of triglycerides and glucose, a simple measure of insulin sensitivity. Comparison with the euglycemic-hyperinsulinemic clamp. J Clin Endocrinol Metab. (2010) 95(7):3347–51. 10.1210/jc.2010-0288 20484475

[9] O'Hagan R Gonzalez-Cantero A Patel N Hong CG Berg AR Li H Association of the triglyceride glucose index with insulin resistance and subclinical atherosclerosis in psoriasis: an observational cohort study. J Am Acad Dermatol. (2023) 88(5):1131–4. 10.1016/j.jaad.2022.08.027 35995090

[10] Liu C Liang D. The association between the triglyceride-glucose index and the risk of cardiovascular disease in US population aged ≤65 years with prediabetes or diabetes: a population-based study. Cardiovasc Diabetol. (2024) 23(1):168. 10.1186/s12933-024-02261-8 38741118 PMC11092030

[11] Tao LC Xu JN Wang TT Hua F Li JJ. Triglyceride-glucose index as a marker in cardiovascular diseases: landscape and limitations. Cardiovasc Diabetol. (2022) 21(1):68. 10.1186/s12933-022-01511-x 35524263 PMC9078015

[12] Hong J Zhang R Tang H Wu S Chen Y Tan X. Comparison of triglyceride glucose index and modified triglyceride glucose indices in predicting cardiovascular diseases incidence among populations with cardiovascular-kidney-metabolic syndrome stages 0-3: a nationwide prospective cohort study. Cardiovasc Diabetol. (2025) 24(1):98. 10.1186/s12933-025-02662-3 40022122 PMC11871812

[13] Zhang P Mo D Zeng W Dai H. Association between triglyceride-glucose related indices and all-cause and cardiovascular mortality among the population with cardiovascular-kidney-metabolic syndrome stage 0-3: a cohort study. Cardiovasc Diabetol. (2025) 24(1):92. 10.1186/s12933-025-02642-7 40022225 PMC11871745

[14] Zhao Y Hu Y Smith JP Strauss J Yang G. Cohort profile: the China health and retirement longitudinal study (CHARLS). Int J Epidemiol. (2014) 43(1):61–8. 10.1093/ije/dys203 23243115 PMC3937970

[15] Inker LA Eneanya ND Coresh J Tighiouart H Wang D Sang Y New creatinine- and cystatin C-based equations to estimate GFR without race. N Engl J Med. (2021) 385(19):1737–49. 10.1056/NEJMoa2102953 34554658 PMC8822996

[16] James PA Oparil S Carter BL Cushman WC Dennison-Himmelfarb C Handler J 2014 evidence-based guideline for the management of high blood pressure in adults: report from the panel members appointed to the eighth joint national committee (JNC 8). J Am Med Assoc. (2014) 311(5):507–20. 10.1001/jama.2013.284427 24352797

[17] Chobanian AV Bakris GL Black HR Cushman WC Green LA Izzo JL Jr The seventh report of the joint national committee on prevention, detection, evaluation, and treatment of high blood pressure: the JNC 7 report. J Am Med Assoc. (2003) 289(19):2560–72. 10.1001/jama.289.19.2560 12748199

[18] American Diabetes Association Diagnosis and classification of diabetes mellitus. Diabetes Care. (2013) 36(Suppl 1):S67–74. 10.2337/dc13-S067 23264425 PMC3537273

[19] Lu Y Zhang H Lu J Ding Q Li X Wang X Prevalence of dyslipidemia and availability of lipid-lowering medications among primary health care settings in China. JAMA Netw Open. (2021) 4(9):e2127573. 10.1001/jamanetworkopen.2021.27573 34586366 PMC8482054

[20] Khan SS Coresh J Pencina MJ Ndumele CE Rangaswami J Chow SL Novel prediction equations for absolute risk assessment of total cardiovascular disease incorporating cardiovascular-kidney-metabolic health: a scientific statement from the American heart association. Circulation. (2023) 148(24):1982–2004. 10.1161/cir.0000000000001191 37947094

[21] Khan SS Matsushita K Sang Y Ballew SH Grams ME Surapaneni A Development and validation of the American heart association’s PREVENT equations. Circulation. (2024) 149(6):430–49. 10.1161/circulationaha.123.067626 37947085 PMC10910659

[22] Li J Lei L Wang W Ding W Yu Y Pu B Social risk profile and cardiovascular-kidney-metabolic syndrome in US adults. J Am Heart Assoc. (2024) 13(16):e034996. 10.1161/jaha.124.034996 39136302 PMC11963957

[23] Ramdas Nayak VK Satheesh P Shenoy MT Kalra S. Triglyceride glucose (TyG) index: a surrogate biomarker of insulin resistance. J Pak Med Assoc. (2022) 72(5):986–8. 10.47391/jpma.22-63 35713073

[24] Park HM Lee HS Lee YJ Lee JH. The triglyceride-glucose index is a more powerful surrogate marker for predicting the prevalence and incidence of type 2 diabetes mellitus than the homeostatic model assessment of insulin resistance. Diabetes Res Clin Pract. (2021) 180:109042. 10.1016/j.diabres.2021.109042 34506839

[25] Wang S Wang Q Yan X. Association between triglyceride-glucose index and hypertension: a cohort study based on the China health and nutrition survey (2009-2015). BMC Cardiovasc Disord. (2024) 24(1):168. 10.1186/s12872-024-03747-9 38504161 PMC10949779

[26] Li Y Gui J Liu H Guo LL Li J Lei Y Predicting metabolic syndrome by obesity- and lipid-related indices in mid-aged and elderly Chinese: a population-based cross-sectional study. Front Endocrinol. (2023) 14:1201132. 10.3389/fendo.2023.1201132 PMC10419183 37576971

[27] Kunutsor SK Seidu S Kurl S Laukkanen JA. Baseline and usual triglyceride-glucose index and the risk of chronic kidney disease: a prospective cohort study. Geroscience. (2024) 46(3):3035–46. 10.1007/s11357-023-01044-5 38180700 PMC11009217

[28] Okamura T Hashimoto Y Hamaguchi M Obora A Kojima T Fukui M. Triglyceride-glucose index is a predictor of incident chronic kidney disease: a population-based longitudinal study. Clin Exp Nephrol. (2019) 23(7):948–55. 10.1007/s10157-019-01729-2 30955189

[29] Pan LY Jin L. Association between triglyceride glucose index and biological aging in U.S. adults: national health and nutrition examination survey. Cardiovasc Diabetol. (2025) 24(1):100. 10.1186/s12933-025-02631-w 40022176 PMC11871761

[30] Tang H Huang J Zhang X Chen X Yang Q Luo N Association between triglyceride glucose-body mass index and the trajectory of cardio-renal-metabolic multimorbidity: insights from multi-state modelling. Cardiovasc Diabetol. (2025) 24(1):133. 10.1186/s12933-025-02693-w 40119385 PMC11929281

[31] Zheng W McLerran DF Rolland B Zhang X Inoue M Matsuo K Association between body-mass index and risk of death in more than 1 million Asians. N Engl J Med. (2011) 364(8):719–29. 10.1056/NEJMoa1010679 21345101 PMC4008249

[32] Huang K Liu F Han X Huang C Huang J Gu D Association of BMI with total mortality and recurrent stroke among stroke patients: a meta-analysis of cohort studies. Atherosclerosis. (2016) 253:94–101. 10.1016/j.atherosclerosis.2016.08.042 27596134

[33] Sarwar N Gao P Seshasai SR Gobin R Kaptoge S Di Angelantonio E Diabetes mellitus, fasting blood glucose concentration, and risk of vascular disease: a collaborative meta-analysis of 102 prospective studies. Lancet. (2010) 375(9733):2215–22. 10.1016/s0140-6736(10)60484-9 20609967 PMC2904878

[34] Gerstein HC. More insights on the dysglycaemia-cardiovascular connection. Lancet. (2010) 375(9733):2195–6. 10.1016/s0140-6736(10)60973-7 20609955

[35] Reaven GM. Banting lecture 1988. Role of insulin resistance in human disease. Diabetes. (1988) 37(12):1595–607. 10.2337/diab.37.12.1595 3056758

[36] Tang WH Kitai T Hazen SL. Gut microbiota in cardiovascular health and disease. Circ Res. (2017) 120(7):1183–96. 10.1161/circresaha.117.309715 28360349 PMC5390330

[37] Sánchez-Íñigo L Navarro-González D Fernández-Montero A Pastrana-Delgado J Martínez JA. The TyG index may predict the development of cardiovascular events. Eur J Clin Invest. (2016) 46(2):189–97. 10.1111/eci.12583 26683265

