# Supplementary material for: The role of triglyceride-glucose index in the progression of cardiovascular-kidney-metabolic syndrome: findings from the China health and retirement longitudinal study
Source: Front Cardiovasc Med. 2025 Jun 23;12:1612121. doi: 10.3389/fcvm.2025.1612121 (PMC12230094; doi:10.3389/fcvm.2025.1612121)
Supplement: Supplementary file 1 [file Table1.docx]

**SUPPLEMENTARY MATERIALS**

**CONTENTS**

**SUPPLEMENTARY MATERIALS1**

**Supplementary Methods3**

Equation for estimated glomerular filtration rate.**3**

Predicted 10-year CVD risk by PREVENT equations.**4**

**Supplementary figures5**

Supplementary figure 1. Association between TyG index and CKM progression...**5**

**Supplementary Tables6**

Supplementary Table 1. Information on missing continuous data.**6**

Supplementary Table 2. Collinearity diagnostics for all covariates in Model 3.**7**

Supplementary Table 3. The HR and 95%CI for the association of TyG index levels and the progress of CKM stages.**8**

Supplementary Table 4. The HR and 95%CI for the association of TyG index levels and the progress of CKM stages among participants with CKM stages 0 and 1 at wave 1.**11**

**Supplementary Methods**

**Equation for estimated glomerular filtration rate**

Estimated glomerular filtration rate (eGFR) was calculated using the chronic kidney disease (CKD) Epidemiology Collaboration (CKD-EPI) 2021 creatinine equation:

eGFR=142×min (standardized SCR/κ, 1)^a^×max (standardized SCR/κ, 1)^-1.200^×0.9938^age^×1.012 (if female)

Abbreviation/ units

eGFR (estimated glomerular filtration rate) = mL/min/1.73 m^2^

SCR (serum creatinine) = mg/dL

κ = 0.7 (females) or 0.9 (males)

a = -0.241 (females) or -0.302 (males)

min = indicates the minimum of SCR/κ or 1

max = indicates the maximum of SCR/κ or 1

**Predicted 10-year CVD risk by PREVENT equations**

| **10-year Total CVD Risk** | |
| --- | --- |
| **Women** | log-Odds = -3.307728 + 0.7939329 × (age – 55) /10 + 0.0305239 × (Tc – HDL – 3.5) – 0.1606857 × (HDL – 1.3) /0.3 – 0.2394003 × (min(SBP, 110) – 110) /20 + 0.360078 × (max(SBP, 110) – 130) /20 + 0.8667604 × (if diabetes) + 0.5360739 × (if current smoker) + 0.6045917 × (min(eGFR, 60) – 60) / -15 + 0.0433769 × (max(eGFR, 60) – 90) / -15 + 0.3151672 × (if using anti-hypertensive medication) – 0.1477655 × (if using statin) – 0.0663612 × (if using anti-hypertensive medication) × (max(SBP, 110) – 130) /20 + 0.1197879 × (if using statin) × (Tc – HDL – 3.5) – 0.0819715 × (age – 55) /10 × (Tc – HDL – 3.5) + 0.0306769 × (age – 55) /10 × (HDL – 1.3) /0.3 – 0.0946348 × (age – 55) /10 × (max(SBP, 110) – 130) /20 – 0.27057 × (age – 55) /10 × (if diabetes) – 0.078715 × (age – 55) /10 × (if current smoker) – 0.1637806 × (age – 55) /10 × (min(eGFR, 60) – 60) / -15  Risk = exp(log-Odds) / (1 + exp(log-Odds)) |
| **Men** | log-Odds = -3.031168 + 0.7688528 × (age – 55) /10 + 0.0736174 × (Tc – HDL – 3.5) – 0.0954431 × (HDL – 1.3) /0.3 – 0.4347345 × (min(SBP, 110) – 110) /20 + 0.3362658 × (max(SBP, 110) – 130) /20 + 0.7692857 × (if diabetes) + 0.4386871 × (if current smoker) + 0.5378979 × (min(eGFR, 60) – 60) / -15 + 0.0164827 × (max(eGFR, 60) – 90) / -15 + 0.288879 × (if using anti-hypertensive medication) – 0.1337349 × (if using statin) – 0.0475924 × (if using anti-hypertensive medication) × (max(SBP, 110) – 130) /20 + 0.150273 × (if using statin) × (Tc – HDL – 3.5) – 0.0517874 × (age – 55) /10 × (Tc – HDL – 3.5) + 0.0191169 × (age – 55) /10 × (HDL – 1.3) /0.3 – 0.1049477 × (age – 55) /10 × (max(SBP, 110) – 130) /20 – 0.2251948 × (age – 55) /10 × (if diabetes) – 0.0895067 × (age – 55) /10 × (if current smoker) – 0.1543702 × (age – 55) /10 × (min(eGFR, 60) – 60) / -15  Risk = exp(log-Odds) / (1 + exp(log-Odds)) |

CVD, cardiovascular disease; TC, total cholesterol; HDL-C, high-density lipoprotein cholesterol; SBP: systolic blood pressure; eGFR, estimated glomerular filtration rate.

**Supplementary figures**

**
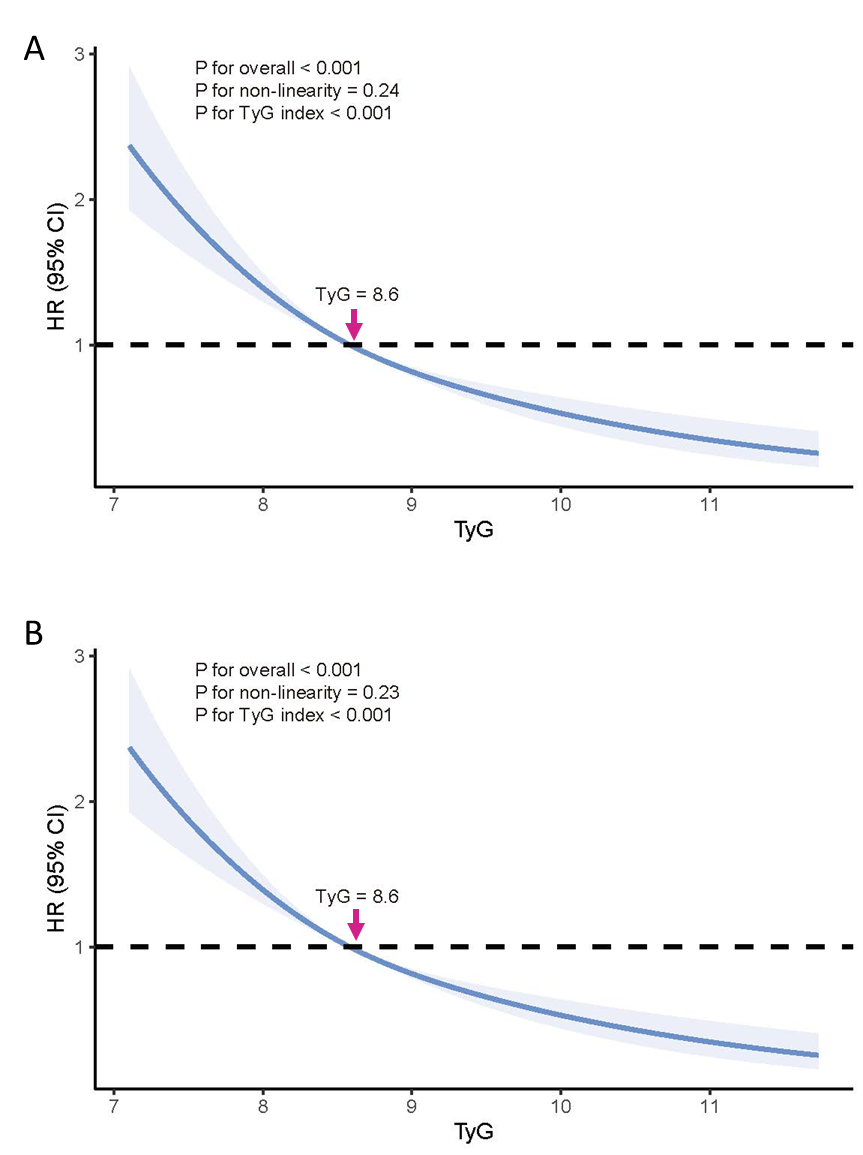
Supplementary figure 1. Association between TyG index and CKM progression**

**Supplementary Tables**

**Supplementary Table 1. Information on missing continuous data.**

| **Variables** | **Missing, n (%)** | **Before imputation** | | **After imputation** | |
| --- | --- | --- | --- | --- | --- |
|  |  | **Mean (SD)** | **Median (IQR)** | **Mean (SD)** | **Median (IQR)** |
| **SCR, mg/dL** | 2 (0.03) | 0.8 (0.2) | 0.7 (0.6, 0.9) | 0.8 (0.2) | 0.7 (0.6, 0.9) |
| **LDL-C, mg/dL** | 3 (0.04) | 115.4 (34.4) | 113.3 (92.4, 135.7) | 115.4 (34.4) | 113.3 (92.4, 135.7) |
| **HbA1c, %** | 44 (7.0) | 5.2 (0.8) | 5.1 (4.9, 5.4) | 5.2 (0.8) | 5.1 (4.9, 5.4) |
| **BMI, kg/m^2^** | 754 (11.9) | 23.9 (13.9) | 23.1 (20.9, 25.7) | 23.8 (13.1) | 23.1 (20.9, 25.7) |
| **WC, cm** | 726 (11.5) | 84.1 (12.3) | 84.2 (77.8, 91.4) | 84.1 (12.2) | 84.2 (77.8, 91.4) |

SCR, serum creatinine; LDL-C, low-density lipoprotein cholesterol; HbA1c, glycosylated hemoglobin; BMI, Body mass index; WC, waist circumference; SD, standard deviations; IQR, interquartile ranges.

**Supplementary Table 2.** Collinearity diagnostics for all covariates in Model 3.

| **Variable** | **VIF** |
| --- | --- |
| TyG | 1.02 |
| Age≥65 | 1.14 |
| Female | 1.78 |
| Married | 1.09 |
| Income | 1.05 |
| Employed | 1.13 |
| Education levels |  |
| Upper secondary & vocational training | 1.06 |
| Tertiary education | 1.03 |
| Rural | 1.07 |
| Smoking | 1.55 |
| Alcohol drinking | 1.27 |
| LDL-C | 1.02 |
| **Mean VIF** | 1.18 |

VIF: variance inflation factors.

**Supplementary Table 3. The HR and 95%CI for the association of TyG index levels and the progress of CKM stages.**

|  | **Model 1** | | **Model 2** | | **Model 3** | |
| --- | --- | --- | --- | --- | --- | --- |
|  | **HR (95% CI)** | **P value** | **HR (95% CI)** | **P value** | **HR (95% CI)** | **P value** |
| **Overall** |  |  |  |  |  |  |
| **TyG (per 1 SD)** | 0.71 (0.67, 0.75) | <0.001 | 0.71 (0.67, 0.75) | <0.001 | 1.03 (0.97, 1.10) | 0.30 |
| **Q1** | Ref. | NA. | Ref. | NA. | Ref. | NA. |
| **Q2** | 0.94 (0.84, 1.05) | 0.29 | 0.95 (0.85, 1.05) | 0.34 | 1.10 (0.98, 1.23) | 0.09 |
| **Q3** | 0.69 (0.61, 0.78) | <0.001 | 0.69 (0.60, 0.78) | <0.001 | 1.10 (0.97, 1.27) | 0.14 |
| **Q4** | 0.45 (0.39, 0.51) | <0.001 | 0.45 (0.39, 0.52) | <0.001 | 1.03 (0.87, 1.22) | 0.71 |
| **Age ≥ 65 years** |  |  |  |  |  |  |
| **TyG (per 1 SD)** | 0.88 (0.80, 0.96) | 0.006 | 0.87 (0.79, 0.95) | 0.003 | 1.10 (0.99, 1.22) | 0.07 |
| **Q1** | Ref. | NA. | Ref. | NA. | Ref. | NA. |
| **Q2** | 0.88 (0.70, 1.11) | 0.28 | 0.88 (0.70, 1.11) | 0.28 | 1.03 (0.81, 1.30) | 0.83 |
| **Q3** | 0.78 (0.63, 0.97) | 0.02 | 0.76 (0.62, 0.95) | 0.01 | 1.06 (0.84, 1.32) | 0.65 |
| **Q4** | 0.78 (0.61, 1.00) | 0.046 | 0.76 (0.60, 0.97) | 0.03 | 1.27 (0.97, 1.67) | 0.09 |
| **Age < 65 years** |  |  |  |  |  |  |
| **TyG (per 1 SD)** | 0.65 (0.61, 0.70) | <0.001 | 0.66 (0.62, 0.70) | <0.001 | 1.07 (0.99, 1.16) | 0.07 |
| **Q1** | Ref. | NA. | Ref. | NA. | Ref. | NA. |
| **Q2** | 0.96 (0.84, 1.09) | 0.53 | 0.96 (0.84, 1.10) | 0.56 | 1.18 (1.03, 1.35) | 0.02 |
| **Q3** | 0.62 (0.53, 0.71) | <0.001 | 0.62 (0.54, 0.72) | <0.001 | 1.18 (1.01, 1.39) | 0.04 |
| **Q4** | 0.34 (0.28, 0.41) | <0.001 | 0.35 (0.29, 0.42) | <0.001 | 1.06 (0.85, 1.33) | 0.59 |
| **Female** |  |  |  |  |  |  |
| **TyG (per 1 SD)** | 0.71 (0.66, 0.77) | <0.001 | 0.71 (0.66, 0.76) | <0.001 | 1.09 (1.00, 1.18) | 0.05 |
| **Q1** | Ref. | NA. | Ref. | NA. | Ref. | NA. |
| **Q2** | 1.08 (0.92, 1.26) | 0.37 | 1.08 (0.92, 1.27) | 0.35 | 1.27 (1.08, 1.50) | 0.004 |
| **Q3** | 0.67 (0.57, 0.79) | <0.001 | 0.67 (0.57, 0.79) | <0.001 | 1.17 (0.98, 1.41) | 0.08 |
| **Q4** | 0.49 (0.40, 0.59) | <0.001 | 0.48 (0.40, 0.59) | <0.001 | 1.23 (0.97, 1.56) | 0.09 |
| **Male** |  |  |  |  |  |  |
| **TyG (per 1 SD)** | 0.71 (0.65, 0.76) | <0.001 | 0.71 (0.66, 0.77) | <0.001 | 0.99 (0.91, 1.08) | 0.84 |
| **Q1** | Ref. | NA. | Ref. | NA. | Ref. | NA. |
| **Q2** | 0.90 (0.76, 1.06) | 0.20 | 0.91 (0.77, 1.08) | 0.28 | 1.02 (0.86, 1.21) | 0.82 |
| **Q3** | 0.74 (0.63, 0.87) | <0.001 | 0.74 (0.63, 0.87) | <0.001 | 1.06 (0.89, 1.26) | 0.53 |
| **Q4** | 0.45 (0.37, 0.55) | <0.001 | 0.46 (0.37, 0.57) | <0.001 | 0.96 (0.76, 1.23) | 0.77 |
| **CKM stage 0** |  |  |  |  |  |  |
| **TyG (per 1 SD)** | 1.05 (0.95, 1.14) | 0.34 | 1.05 (0.96, 1.15) | 0.30 | 1.04 (0.95, 1.14) | 0.41 |
| **Q1** | Ref. | NA. | Ref. | NA. | Ref. | NA. |
| **Q2** | 0.89 (0.69, 1.16) | 0.41 | 0.89 (0.68, 1.16) | 0.40 | 0.89 (0.68, 1.17) | 0.41 |
| **Q3** | 0.96 (0.76, 1.21) | 0.72 | 0.95 (0.75, 1.21) | 0.70 | 0.94 (0.74, 1.20) | 0.64 |
| **Q4** | 1.06 (0.84, 1.34) | 0.62 | 1.07 (0.85, 1.35) | 0.58 | 1.05 (0.83, 1.34) | 0.67 |
| **CKM stage 1** |  |  |  |  |  |  |
| **TyG (per 1 SD)** | 1.17 (1.07, 1.27) | <0.001 | 1.17 (1.08, 1.27) | <0.001 | 1.15 (1.05, 1.25) | 0.002 |
| **Q1** | Ref. | NA. | Ref. | NA. | Ref. | NA. |
| **Q2** | 1.03 (0.80, 1.32) | 0.83 | 1.03 (0.80, 1.32) | 0.81 | 1.00 (0.78, 1.29) | 0.96 |
| **Q3** | 1.30 (1.04, 1.64) | 0.02 | 1.30 (1.04, 1.64) | 0.02 | 1.27 (1.00, 1.60) | 0.046 |
| **Q4** | 1.39 (1.12, 1.71) | 0.002 | 1.39 (1.12, 1.72) | 0.002 | 1.34 (1.08, 1.66) | 0.008 |
| **CKM stage 2** |  |  |  |  |  |  |
| **TyG (per 1 SD)** | 1.02 (0.95, 1.10) | 0.59 | 1.02 (0.95, 1.10) | 0.56 | 1.02 (0.95, 1.10) | 0.57 |
| **Q1** | Ref. | NA. | Ref. | NA. | Ref. | NA. |
| **Q2** | 1.13 (0.93, 1.38) | 0.22 | 1.14 (0.93, 1.39) | 0.20 | 1.14 (0.93, 1.40) | 0.20 |
| **Q3** | 0.96 (0.78, 1.19) | 0.72 | 0.95 (0.77, 1.16) | 0.60 | 0.95 (0.77, 1.17) | 0.62 |
| **Q4** | 1.18 (0.96, 1.44) | 0.12 | 1.17 (0.96, 1.44) | 0.13 | 1.17 (0.96, 1.44) | 0.13 |
| **CKM stage 3** |  |  |  |  |  |  |
| **TyG (per 1 SD)** | 0.92 (0.65, 1.28) | 0.61 | 0.95 (0.67, 1.34) | 0.77 | 0.93 (0.66, 1.32) | 0.69 |
| **Q1** | Ref. | NA. | Ref. | NA. | Ref. | NA. |
| **Q2** | 0.78 (0.35, 1.70) | 0.53 | 0.83 (0.37, 1.83) | 0.64 | 0.81 (0.37, 1.80) | 0.61 |
| **Q3** | 0.84 (0.39, 1.79) | 0.65 | 0.86 (0.40, 1.85) | 0.69 | 0.84 (0.39, 1.83) | 0.66 |
| **Q4** | 1.02 (0.44, 2.35) | 0.97 | 1.08 (0.46, 2.51) | 0.87 | 1.03 (0.44, 2.43) | 0.94 |

TyG: Triglyceride-Glucose index; CKM: cardiovascular-kidney-metabolic syndrome. Data were presented as number (percentage); SD; HR: hazard ratio, CI confidence intervals.

Multivariable Cox proportional hazards regression models were employed to estimate hazard ratio and 95% confidence intervals for the association between TyG index levels and CKM progression.

Model 1: adjusted for age and gender.

Model 2: Model 1, marriage status, annuals household income, occupation status, education levels, and urbanity.

Model 3: Model 2, CKM stage at wave 1, smoking, alcohol drinking, low-density lipoprotein cholesterol.

**Supplementary Table 4. The HR and 95%CI for the association of TyG index levels and the progress of CKM stages among participants with CKM stages 0 and 1 at wave 1.**

| **Different types of CKM progression** | **CKM stage 0 at wave 1** | | | **CKM stage 1 at wave 1** | | |
| --- | --- | --- | --- | --- | --- | --- |
|  | **Model 1** | **Model 2** | **Model 3** | **Model 1** | **Model 2** | **Model 3** |
| **CKD** |  |  |  |  |  |  |
| **Q1** | Ref. | Ref. | Ref. | Ref. | Ref. | Ref. |
| **Q2** | 0.66 (0.06, 7.24) | 1.11 (0.08, 14.56) | 1.25 (0.07, 21.60) | 2.48 (0.93, 6.62) | 2.19 (0.81, 5.91) | 2.39 (0.85, 6.70) |
| **Q3** | 1.47 (0.25, 8.82) | 1.84 (0.28, 12.03) | 3.24 (0.38, 27.77) | 1.71 (0.61, 4.82) | 1.70 (0.60, 4.82) | 2.00 (0.68, 5.88) |
| **Q4** | 2.34 (0.45, 12.11) | 2.30 (0.38, 13.91) | 2.87 (0.27, 30.28) | 1.65 (0.62, 4.40) | 1.55 (0.58, 4.16) | 1.80 (0.63, 5.13) |
| **Clinical CVD** |  |  |  |  |  |  |
| **Q1** | Ref. | Ref. | Ref. | Ref. | Ref. | Ref. |
| **Q2** | 0.63 (0.22, 1.82) | 0.69 (0.23, 2.00) | 0.67 (0.22, 1.99) | 1.01 (0.51, 1.98) | 0.87 (0.44, 1.73) | 0.84 (0.42, 1.69) |
| **Q3** | 0.45 (0.16, 1.29) | 0.43 (0.15, 1.26) | 0.36 (0.12, 1.08) | 1.59 (0.88, 2.90) | 1.41 (0.77, 2.58) | 1.40 (0.75, 2.62) |
| **Q4** | 1.18 (0.53, 2.65) | 1.35 (0.59, 3.07) | 1.10 (0.45, 2.68) | 0.87 (0.46, 1.64) | 0.76 (0.40, 1.46) | 0.71 (0.36, 1.40) |
| **CKM stage at wave 3 - CKM stage at wave 1 ≥2** | | | | | | |
| **Q1** | Ref. | Ref. | Ref. | Ref. | Ref. | Ref. |
| **Q2** | 1.01 (0.68, 1.48) | 1.04 (0.70, 1.54) | 1.06 (0.71, 1.58) | 1.09 (0.64, 1.87) | 0.93 (0.54, 1.61) | 0.85 (0.49, 1.48) |
| **Q3** | 1.06 (0.75, 1.51) | 1.11 (0.78, 1.58) | 1.04 (0.73, 1.49) | 1.39 (0.85, 2.29) | 1.14 (0.69, 1.88) | 1.12 (0.67, 1.88) |
| **Q4** | 1.40 (1.01, 1.94) ^*^ | 1.46 (1.04, 2.04) ^*^ | 1.32 (0.93, 1.89) | 1.06 (0.65, 1.73) | 0.88 (0.54, 1.46) | 0.77 (0.45, 1.30) |
| **Q4** | 0.80 (0.57, 1.11) | 0.79 (0.56, 1.11) | 0.77 (0.54, 1.10) | 1.49 (1.18, 1.88) ^***^ | 1.54 (1.22, 1.95) ^***^ | 1.40 (1.09, 1.79) ^**^ |

TyG: Triglyceride-Glucose index; CKM: cardiovascular-kidney-metabolic syndrome. Data were presented as number (percentage); SD; HR: hazard ratio, CI confidence intervals.

Cox proportional hazards regression models were employed to estimate hazard ratio and 95% confidence intervals for the association between TyG index levels and CKM progression. Model 1: non-adjusted; Model 2: adjusted for age, gender, marriage status, annuals household income, occupation status, education levels, urbanity, smoking, alcohol drinking; Model 3: adjusted for for age, gender, marriage status, annuals household income, occupation status, education levels, urbanity, smoking, alcohol drinking, low-density lipoprotein cholesterol, eGFR, BMI, waist, HbA1c, HDL-C, SBP, and DBP.

^***^, *P* < 0.001; ^**^, *P* < 0.01; ^*^, *P* < 0.05.
